# An integrated study of glutamine alleviates enteritis induced by glycinin in hybrid groupers using transcriptomics, proteomics and microRNA analyses

**DOI:** 10.3389/fimmu.2023.1301033

**Published:** 2023-11-22

**Authors:** Yuanfa He, Xiaohui Dong, Qihui Yang, Hongyu Liu, Shuang Zhang, Shiwei Xie, Shuyan Chi, Beiping Tan

**Affiliations:** ^1^ Laboratory of Aquatic Animal Nutrition and Feed, College of Fisheries, Guangdong Ocean University, Zhanjiang, China; ^2^ College of Fisheries, Southwest University, Chongqing, China; ^3^ Key Laboratory of Aquatic, Livestock and Poultry Feed Science and Technology in South China, Ministry of Agriculture and Rural Affair, Zhanjiang, China

**Keywords:** amino acid, anti-nutritional factor, intestinal inflammation, multi-omics, signaling pathway

## Abstract

Glutamine has been used to improve intestinal development and immunity in fish. We previously found that dietary glutamine enhances growth and alleviates enteritis in juvenile hybrid groupers (*Epinephelus fuscoguttatus♀ × Epinephelus lanceolatus♂*). This study aimed to further reveal the protective role of glutamine on glycinin-induced enteritis by integrating transcriptome, proteome, and microRNA analyses. Three isonitrogenous and isolipidic trial diets were formulated: a diet containing 10% glycinin (11S group), 10% glycinin diet supplemented with 2% alanine-glutamine (Gln group), and a diet containing neither glycinin nor alanine-glutamine (fishmeal, FM group). Each experimental diet was fed to triplicate hybrid grouper groups for 8 weeks. The analysis of intestinal transcriptomic and proteomics revealed a total of 570 differentially expressed genes (DEGs) and 169 differentially expressed proteins (DEPs) in the 11S and FM comparison group. Similarly, a total of 626 DEGs and 165 DEPs were identified in the Gln and 11S comparison group. Integration of transcriptome and proteome showed that 117 DEGs showed consistent expression patterns at both the transcriptional and translational levels in the Gln and 11S comparison group. These DEGs showed significant enrichment in pathways associated with intestinal epithelial barrier function, such as extracellular matrix (ECM)-receptor interaction, tight junction, and cell adhesion molecules (*P* < 0.05). Further, the expression levels of genes (*myosin-11*, *cortactin*, *tenascin*, *major histocompatibility complex class I* and *II*) related to these pathways above were significantly upregulated at both the transcriptional and translational levels (*P* < 0.05). The microRNA results showed that the expression levels of miR-212 (target genes *colla1* and *colla2*) and miR-18a-5p (target gene *colla1*) in fish fed Gln group were significantly lower compared to the 11S group fish (*P* < 0.05). In conclusion, ECM-receptor interaction, tight junction, and cell adhesion molecules pathways play a key role in glutamine alleviation of hybrid grouper enteritis induced by high-dose glycinin, in which miRNAs and target mRNAs/proteins participated cooperatively. Our findings provide valuable insights into the RNAs and protein profiles, contributing to a deeper understanding of the underlying mechanism for fish enteritis.

## Introduction

Soya glycinin, accounting for 40% of the total protein in soy seed, has been identified as a major anti-nutritional factor and has a hexameric structure consisting of six subunits with the basic structure A-S-S-B (disulfide bond, where A and B represent the acidic and basic subunits, respectively) (1). Its antigenicities are relatively stable and are not easily destroyed at 100°C temperature treatment. Nowadays, the methods of mitigating soya glycinin-induced enteritis or antigenicity are physical (2), chemical (3), biological (4), and the application of innovative feed additives (5). However, in-depth research is still needed to completely remove the immunogenicity of soya glycinin. High-dose glycinin can impair intestinal immune function, cause inflammation response, and ultimately inhibit growth performance in fish (5–8). In general, soya glycinin-induced intestinal inflammation is accompanied by mRNA levels of *zonula occludin-1* (*zo-1*), *occludin* and *claudin-4* reduced as well as *interleukin-1β* and *tumor necrosis factor-α* increased (5, 9). Transcriptomic techniques have been employed to investigate the differential expression of soybean meal-induced enteritis (SBMIE) and affect its immune system-related pathways including cytokine-cytokine receptor interactions, intestinal immune network for immunoglobulin A (IgA) production, nuclear factor NF-κB signaling pathway, Jak (Janus kinase)-STAT (Signal transducers and activators of transcription) signaling pathway, T-cell receptor signaling pathway and tumor necrosis factor (TNF) signaling pathway, which play key roles in responding to soybean meal stress in fish (10, 11). The utilization of proteomics has provided valuable insights into the intricate molecular mechanisms by which fish respond to external stimuli such as feed additives. The influences of dietary tryptophan on the growth and physiology of snapper (*Sparus aurata*) were studied previously, and its proteomic data showed that dietary tryptophan did not affect growth but stimulated immunity in the fish (12). However, the integrated transcriptomic and proteomics analyses have been less studied in fish, and the integration of transcriptomic and proteomics analysis can provide more complete information compared to single omics, as well as the two can mutually validate the reliability of the data.

Although transcriptomic and proteomics technologies can provide a comprehensive understanding of overall molecular level changes, inconsistent expression levels may exist between mRNAs and proteins (13, 14). In addition to deficiencies in high-throughput omics technology and incompleteness of mRNA/protein databases, the complex regulatory mechanisms underlying the translation of mRNAs into mature proteins may also lead to inconsistent results. MicroRNAs (miRNAs) are major regulators of cellular function (15), prominently contributing to post-transcriptional and translational gene expression through various mechanisms (16). In addition, miRNAs have been found to have important roles in regulating intestinal functions such as epithelial cell growth (17), mucosal barrier function (18), and the development of gastrointestinal disease (19–21). As an important aspect, mRNA expression levels in fish can be regulated by miRNA targeting. A miRNAome study on the intestinal immune function of turbot (*Scophthalmus maximus* L.) showed that differentially expressed miRNAs contribute to the enhancement of intestinal immune response and the prevention of host infection, where their target genes are implicated in diverse immune functions and inflammatory responses (22). Meanwhile, fish composition influences the expression levels of intestinal miRNAs and their target genes, as well as some pathways, such as cell adhesion molecules, ECM-receptor interaction, apoptotic signaling pathway, and cytokine-cytokine receptor interaction, were identified by small RNA sequencing (11, 23).

The nutritional strategies of feed additives for aquatic animals have been studied separately at the mRNA, protein, or miRNA molecules. However, these molecules are interconnected and can mutually influence. mRNAs are transcribed from genes and act as templates for protein synthesis, while miRNAs exert regulatory control over protein translation or mRNA stability (24). This genetic information flow ultimately leads to the synthesis of proteins, which play various roles in biological processes. Thus, the integration of these three components (mRNAs, proteins, and miRNAs) is essential for a comprehensive study of fish intestinal health.

The national production of grouper is at 205,816 tons in 2022, which has become the third most productive species among marine economic fish species (25). Our previous study reported that the addition of purified high-dose glycinin to the diet reduced growth performance and caused enteritis in juvenile hybrid groupers (*Epinephelus fuscoguttatus*♀ × *Epinephelus lanceolatus*♂) (26). We also found that feed supplementation with 2% alanyl-glutamine enhanced growth performance and alleviated enteritis induced by glycinin in the same species (27). However, the potential protective mechanisms for glutamine to alleviate enteritis in fish based on multi-omics techniques have not been studied. This experiment aimed to further reveal the potential protective role of glutamine (Gln) against glycinin-induced enteritis in hybrid groupers by integrating transcriptomics, proteomics, and miRNA analyses. In addition, because Gln tends to become hot and less soluble during feed processing, a feed substitute for Gln, alanyl-glutamine, was used for the study (28–30).

## Materials and methods

### Grouping and sample collection

Three experimental diets were prepared with equal levels of protein (48% crude protein) and lipid (12% crude lipid): a diet based on fishmeal (referred to as Group FM), a diet containing 10% glycinin (referred to as Group 11S), and glycinin diet supplemented with 2% alanine-glutamine (referred to as Group Gln). The feed formulation is based on our published articles (24). Juvenile hybrid groupers used in this experiment were obtained from a local commercial hatchery (Zhanjiang, Guangdong, China). Healthy and vigorous hybrid groupers (8.50 ± 0.01 g) were fed each diet for 8 weeks. After the feeding trial finished, distal intestine (DI) samples from the three groups were obtained to determine transcriptome, proteome, and miRNA levels.

### Transcriptome sequencing and *de novo* assembly

A total of 1 μg of RNA from FM, 11S, and Gln experimental groups was utilized for the preparation of transcriptome library. Initial steps involved the generation of first-strand cDNA through PCR, followed by the subsequent generation of second-strand cDNA. Subsequent to PCR amplification of cDNA fragments along with adapters, the resulting products underwent purification using AMPure XP Beads. Subsequently, the purified double-stranded cDNA underwent end-repaired, A-tailing, and ligation to sequencing junctions. Ultimately, PCR enrichment yielded the final cDNA library. The library’s quality was then assessed using the Agilent Technologies 2100 bioanalyzer, followed by sequencing on the Illumina platform. Raw data underwent filtration to eliminate adapter sequences and low-quality reads, resulting in a collection of high-quality clean reads, which was assembled to obtain a Unigene library for the species. Once high-quality sequencing data has been obtained, it needs to be assembled using Trinity software (31). Trinity-derived transcripts served as reference sequences (Ref), against which clean reads from each sample were aligned and compared. Finally, reliable transcripts were obtained by filtering the low-expression transcripts. Following the assembly process, the assembled All-Unigenes were subjected to comprehensive annotation against the publicly accessible protein databases, which encompassed GO (Gene Ontology), KOG (EuKaryotic Orthologous Groups), Swiss-prot, Nt (non-redundant nucleotide sequences), and Nr (non-redundant protein sequences). The quantification of gene expression level relied on the expected number of fragments per kilobase of transcript per million mapped reads (FPKM). Differentially expressed genes (DEGs) between the two groups were pinpointed using a criterion of fold change (FC) ≥ 1.5 and a false discovery rate (FDR) of < 0.05. The process of pathway assignments involved utilizing sequences to query the KEGG database, with KEGG terms having corrected *P*-values (*Q*-values) of ≤ 0.05 deemed significant. Transcriptome (*de novo* assembly) sequencing data have been *submitted to* the NCBI SRA database with the accession number PRJNA1008292.

### Proteome sequencing and analysis

The quantitative proteomic analysis of gut tissues from hybrid groupers was carried out using a 4D-label-free approach at Jingjie PTM Biolabs Inc. (Hangzhou, China). As previously described by Jiang et al. (32), the gut samples were initially ground, lysed, and subjected to centrifugation to yield the supernatant. The protein concentration of the supernatant was measured. Following trichloroacetic acid precipitation and acetone washing, protein samples were dissolved in triethylamine borane and digested with trypsin to yield peptides. Subsequently, peptides were desalted through Strata X SPE column, separated using NanoElute ultra-high-performance liquid system, and introduced into the capillary ion source for ionization. The mass spectrometry analysis was carried out using the timsTOF Pro (tims: trapped ion mobility spectrometry; TOF: time of flight) manufactured by Bruker in United States.

We employed the Maxquant search engine (v1.6.15.0) to process raw data from mass spectrometry. The transcriptome database of hybrid grouper (fasta format) was utilized as a reverse decoy database to facilitate the identification of matching proteins from the tandem mass spectra. Additionally, a reverse database was integrated to estimate the false discovery rate (FDR) resulting from random matches. Contaminated proteins within the identified list were excluded to minimize their impact. Cleavage enzyme specificity was designated as Trypsin/P, allowing for a maximum of 2 missing cleavages. Peptides were required to have a minimum length of seven amino acid residues, and a maximum of 5 modifications were considered. Precursor ion mass tolerance was set as 20 ppm for both the First search and Main search phases. Similarly, a mass tolerance of 20 ppm was applied to fragment ions. Fixed modifications encompassed carbamidomethyl on cysteine, while variable modifications encompassed methionine oxidation and protein N-terminal acetylation. To ensure robust identification quality, an FDR of 1% was maintained for protein and peptide identification. Differential proteins were identified after sample qualification. Their relative quantification differences between the two groups were assessed through a T-test, yielding the corresponding p-value. Furthermore, utilizing a p-value criterion of ≤ 0.05, protein ratios exceeding 1.2 was considered up-regulated, while a ratio less than 1/1.2 was considered down-regulated. Using the list of identified proteins, we conducted a subcellular localization analysis through the WoLF-PSORT database. The pathway analysis was executed utilizing the KEGG database. Furthermore, we employed a two-tailed test to analyze enriched pathways and ascertain the enrichment of differentially expressed proteins. A significance threshold of *P*-value ≤ 0.05 was applied. The MS proteomics data have been *submitted to* the ProteomeXchange Consortium via the iProX partner repository with the dataset identifier PXD044757.

### miRNA qPCR analysis

Screening of miRNAs regulating key genes associated with the intestinal barrier pathways based on a small RNA sequencing database in hybrid groupers. Small RNA transcriptome data were submitted to the SRA database under the accession number SUB7175134. Isolation of miRNA from the intestinal tract was conducted utilizing the RNAiso from small RNA Kit (Takara, China). Subsequently, mature miRNA’s first-strand cDNA was conducted using the Mir-X™ miRNA First-Strand Synthesis Kit (Takara, China). Quantitative analysis used the miRNA SYBR Green RT-qPCR Kit (Takara, China) with the provided miRNA reference gene (U6). The specific primers for the target miRNA used in this study are detailed in **Supplementary Table 1**. Relative quantitative was determined by the 2^-ΔΔCT^ method (33).

### Transcriptome and proteome validation

The identical samples employed for transcriptome analysis underwent RT-qPCR validation (n *= 3). Primers were designed using Premier 5.0 and subsequently validated using the online Primer-BLAST program. Primer sequences are provided in* **Supplementary Table 1**. For mRNA sequencing, 1 μg of RNA was subjected to reverse transcribed to generate cDNA. Real-time PCR assays were conducted using the CFX96 real-time PCR Detection System. The reference gene β-Actin was chosen based on a prior study (34). Similarly, relative quantitative was determined by the 2^-ΔΔCT^ method (33).

Protein abundance levels were validated through the quantification of eight selected proteins using parallel reaction monitoring-mass spectrometry (PRM-MS) analysis conducted by Jingjie PTM BioLab Co., Ltd. (Hangzhou, China). Relative quantification using the PRM approach was employed, utilizing signature peptides derived from the target proteins identified based on the 4D-label-free data. Quantification was established with a minimum peptide count of 2, encompassing both unique and razor peptides. Protein extraction and trypsin digestion were conducted as previously outlined. Following the approach outlined in the earlier study (35), peptides were dissolved and then subjected to tandem mass spectrometry in conjunction with liquid chromatography (LC-MS/MS). Subsequently, the acquired MS data underwent processing utilizing Skyline software (v.3.6), which included the setting of several parameters.

### Statistics analysis

Analysis of miRNA expression level was evaluated using a two-tailed t-test (GraphPad Prism 8.0). For significant differences, * 0.01<*P*<0.05 and ** 0.001<*P*<0.01 between the two groups. The software GraphPad Prism 8.0 was used to generate the histograms.

## Results

### mRNA sequencing analysis

A total of nine qualified libraries were subjected to sequencing, distributed across the FM, 11S, and Gln groups, with each group consisting of three biological replicates. **Table 1** provides a concise overview of the sequencing and assembly details. The FM, 11S, and Gln groups yielded approximately 19.94, 18.05, and 18.26 Gb of clean reads, respectively. Over 91.72% of the reads exhibited Q-scores at the Q30 level, and over 63.22% of the clean reads were successfully aligned.

**Table 1:** Overview of mRNA sequencing datasets from the intestine (9 samples).

Data type	BaseSum	GC(%)	Q20(%)	Q30(%)	Total_Reads	Mapped_Reads	Mapped_Ratio
FM_1	6,284,606,100	43.59%	96.34%	91.12%	20948687	13278452	63.39%
FM_2	6,619,536,600	42.13%	96.67%	91.89%	22065122	14276743	64.70%
FM_3	6,590,311,200	42.01%	96.20%	90.99%	21967704	13669264	62.22%
11S_1	6,591,993,000	43.58%	96.98%	92.27%	21973310	14859914	67.63%
11S_2	5,596,750,200	44.06%	97.32%	92.99%	18655834	12999383	69.68%
11S_3	5,859,667,500	43.58%	96.88%	92.37%	20362363	13610968	66.84%
Gln_1	5,801,036,700	42.44%	96.39%	91.31%	19336789	12502238	64.66%
Gln_2	6,165,045,300	42.18%	96.69%	92.04%	20550151	12499831	60.83%
Gln_3	6,289,937,700	41.07%	95.87%	90.48%	20966459	10283714	49.05%

### Differentially expressed genes and KEGG pathway analysis

**Figure 1:**
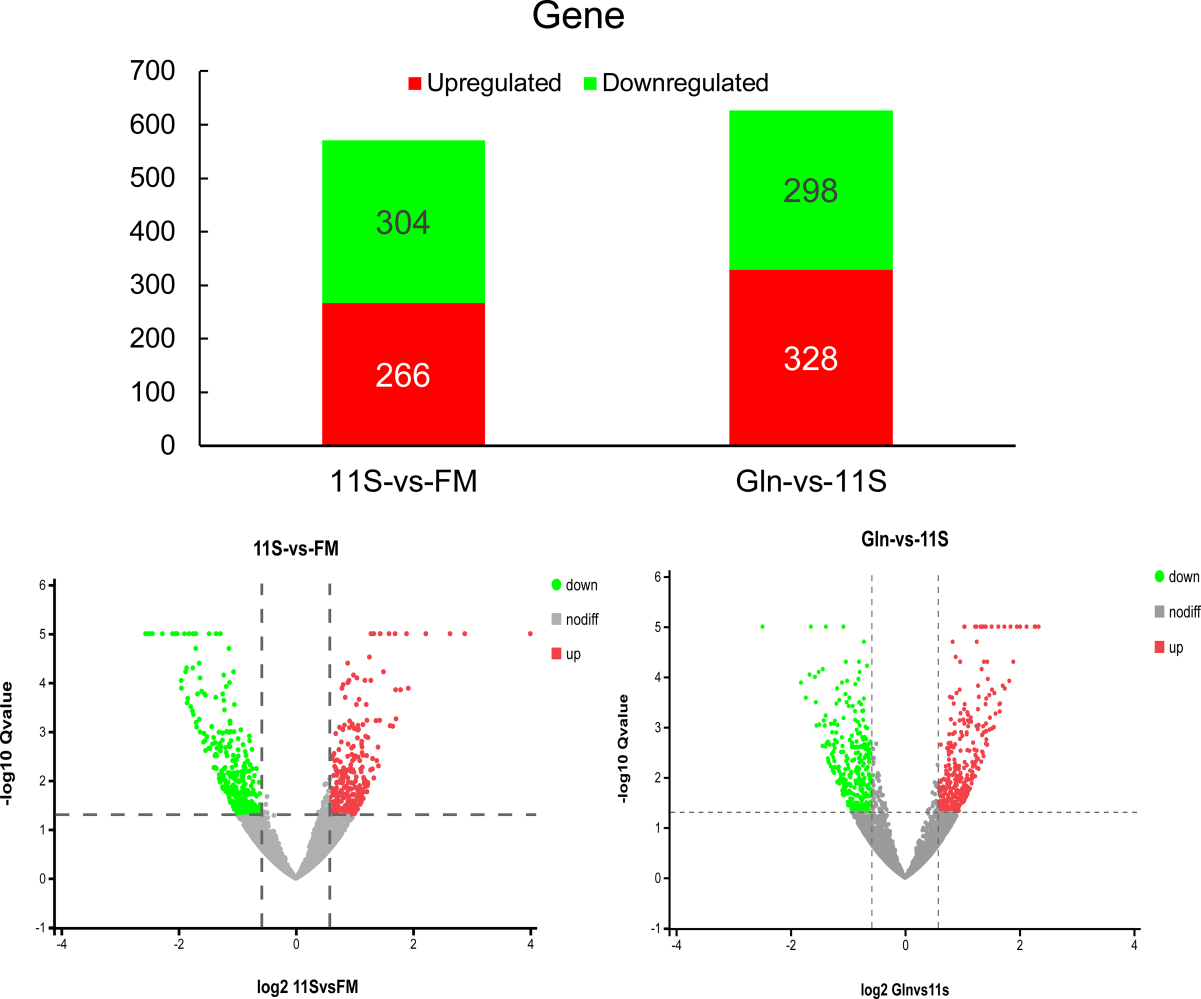
Analysis of histogram and volcano plot of differentially expressed genes (DEGs) in both 11S-vs-FM and Gln-vs-11S comparison groups. The horizontal and vertical axis in the volcano plot represents the DEGs value and -log10 p-value, respectively. In this visualization, upregulated DEGs are marked by red dots. Conversely, downregulated DEGs are indicated by green dots. Genes displaying no significant difference in expression are marked by gray dots.

As shown in **Figure 1**, 570 DEGs were identified in the 11S and FM comparison group, with 266 upregulation and 304 downregulation genes (FC > 1.5). Likewise, 626 DEGs were identified in the Gln and 11S comparison group, with 328 upregulation and 298 downregulation genes. Principal component analysis (PCA) was used to assess the similarities within samples and whether the samples could be grouped well (**Supplementary Figure 1**).

**Figure 2:**
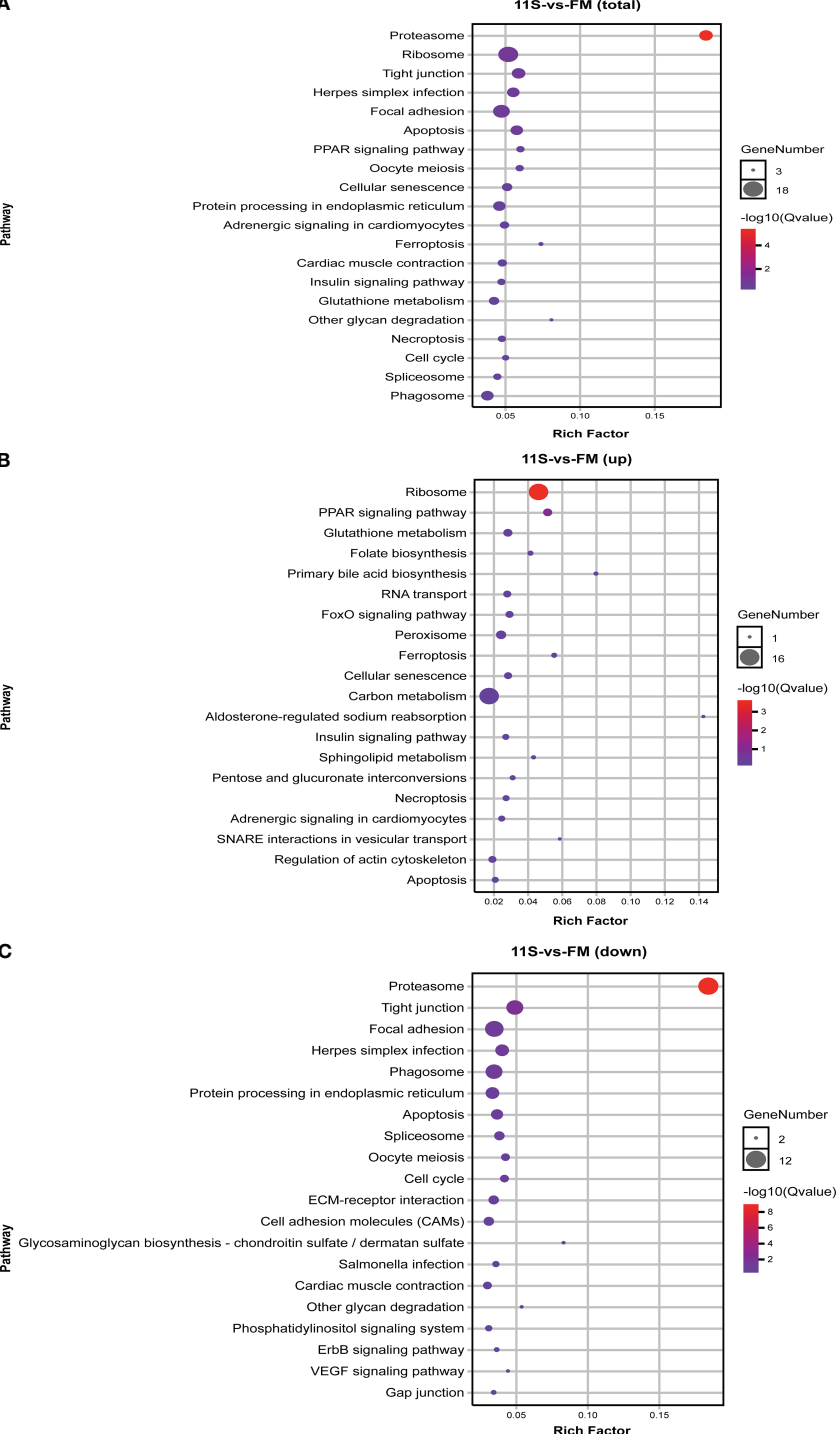
KEGG pathways enrichment analysis for differentially expressed genes (DEGs) in 11S-vs-FM comparison group. **(A)** Enrichment by total DEGs. **(B)** Enrichment by upregulated DEGs. **(C)** Enrichment by downregulated DEGs.

In the comparison of 11S and FM groups, 570 DEGs were enriched in 153 pathways, with the counts of DEGs within each enriched pathway ranging from 3 to 18 (**Figure 2A**). Among these, the prominent KEGG pathways comprised immune system- and human disease-related pathways such as phagosome (ko04145) and herpes simplex infection (ko05168), and intestinal epithelial barrier-related pathways such as tight junction (ko04530) and focal adhesion (ko04510). Additionally, pathways linked to cell growth and death, such as apoptosis (ko04215) and necroptosis (k04217) were also significantly enriched (P *< 0.05*, **Figure 2A**). Furthermore, upregulated genes showed significant enrichment in ribosome (ko03010), PPAR signaling pathway (ko03320), ferroptosis (ko04216), primary bile acid biosynthesis (ko00120), glutathione metabolism (ko00480), FoxO signaling pathway (ko04068) and peroxisome (ko04146, P *< 0.05*, **Figure 2B**). Downregulated genes showed significant enrichment in intestinal epithelial barrier-related pathways such as ECM-receptor interaction (ko04512), tight junction, focal adhesion, and cell adhesion molecules (ko04514; P *< 0.05*, **Figure 2C**).

**Figure 3:**
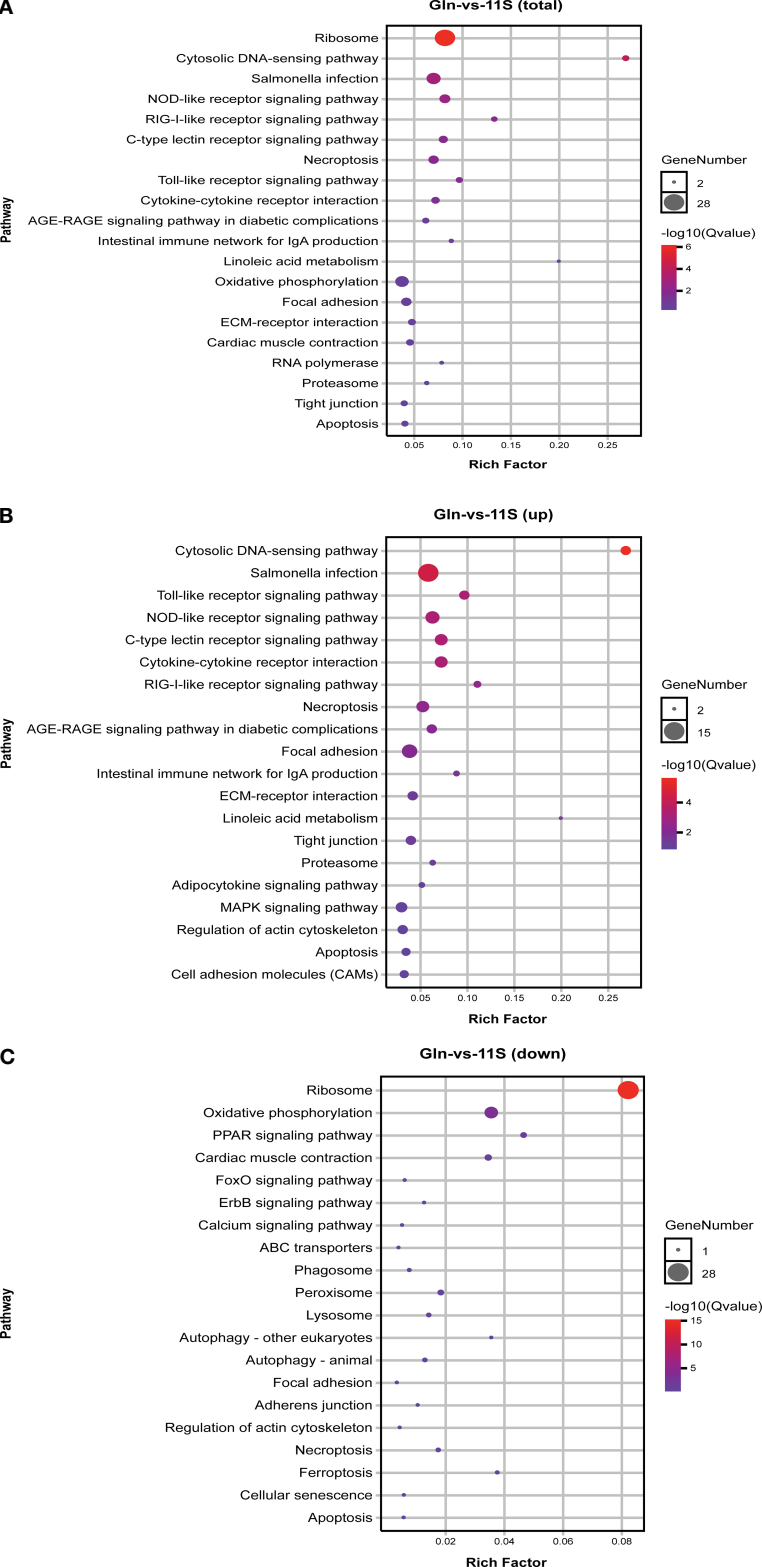
KEGG pathways enrichment analysis for differentially expressed genes (DEGs) in Gln-vs-11S comparison group. **(A)** Enrichment by total DEGs. **(B)** Enrichment by upregulated DEGs. **(C)** Enrichment by downregulated DEGs.

In the comparison of Gln and 11S groups, 626 DEGs were enriched in 133 pathways, with the counts of DEGs within each enriched pathway ranging from 2 to 28 (**Figure 3A**). Among them, the leading 20 KEGG pathways showed significant enrichment in immune system- and human disease-related pathways, including NOD-like receptor signaling pathway (ko04621), C-type lectin receptor signaling pathway (ko04625), RIG-I-like receptor signaling pathway (ko04622), intestinal immune network for IgA production (ko04672), toll-like receptor signaling pathway (ko04620), salmonella infection (ko05132) and cardiac muscle contraction (ko04260). Additionally, pathways associated with intestinal epithelial barriers, including tight junction, focal adhesion and ECM-receptor interaction (ko04512), were also notably enriched (P *< 0.05*, **Figure 3A**). Upregulated genes showed significant enrichment in pathways linked to the immune system, including toll-like receptor signaling pathway, NOD-like receptor signaling pathway, C-type lectin receptor signaling pathway, RIG-I-like receptor signaling pathway, intestinal immune network for IgA production and MAPK signaling pathway (ko04010; P *< 0.05*, **Figure 3B**). Additionally, they were significantly enriched in intestinal epithelial barrier-related pathways such as focal adhesion, regulation of actin cytoskeleton, ECM-receptor interaction, tight junction, and cell adhesion molecules (CAMs; P *< 0.05). Downregulated genes showed significant enrichment in the ribosome, oxidative phosphorylation (ko00190)*, PPAR signaling pathway (ko03320), and cardiac muscle contraction (ko04260; P *< 0.05*, **Figure 3C**).

### Differentially expressed proteins and subcellular localization analysis

**Figure 4:**
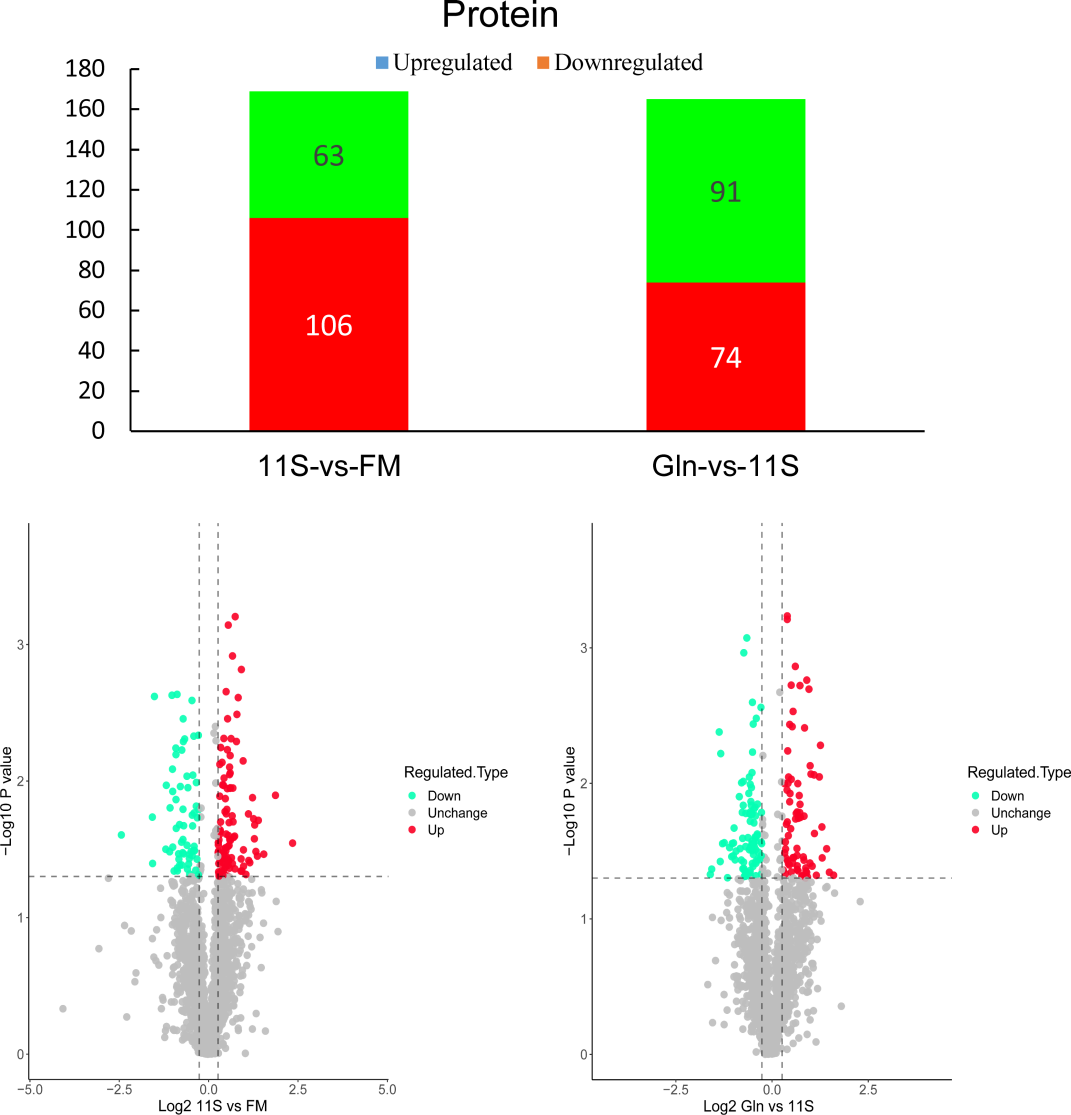
Analysis of histogram and volcano plot of differentially expressed proteins (DEPs) in both 11S-vs-FM and Gln-vs-11S comparison groups. The horizontal and vertical axis in the volcano plot represents the DEPs value and -log10 p-value, respectively. In this visualization, upregulated DEPs are denoted by red dots. Conversely, downregulated DEPs are denoted by green dots. Proteins displaying no significant difference in expression are marked by gray dots.

As shown in **Figure 4**, a total of 169 DEPs were found in the 11S and FM comparison group, with 106 upregulated proteins and 63 downregulated proteins (FC >1.2). A total of 165 DEPs were found in the Gln and 11S comparison group, including 74 upregulated proteins and 91 downregulated proteins. Subcellular localization analysis showed that 78 proteins were situated in the cytoplasm (46.15%, **Supplementary Figure 2A**); 32 proteins were located in the mitochondria (18.93%); 19 proteins were localized in extracellular (11.24%); 17 proteins were localized in the nucleus (10.06%). Similarly, of the 165 DEPs in the Gln and 11S comparison group: 77 proteins were localized in the cytoplasm (46.67%); 32 proteins were localized in the mitochondria (19.39%); 19 proteins were localized in the nucleus (11.52%); 10 proteins were localized in the plasma membrane (6.06%); as well as 8 proteins were localized in the cytoplasm and nucleus (4.85%; **Supplementary Figure 2B**).

### KEGG pathway analysis for differentially expressed proteins

**Figure 5:**
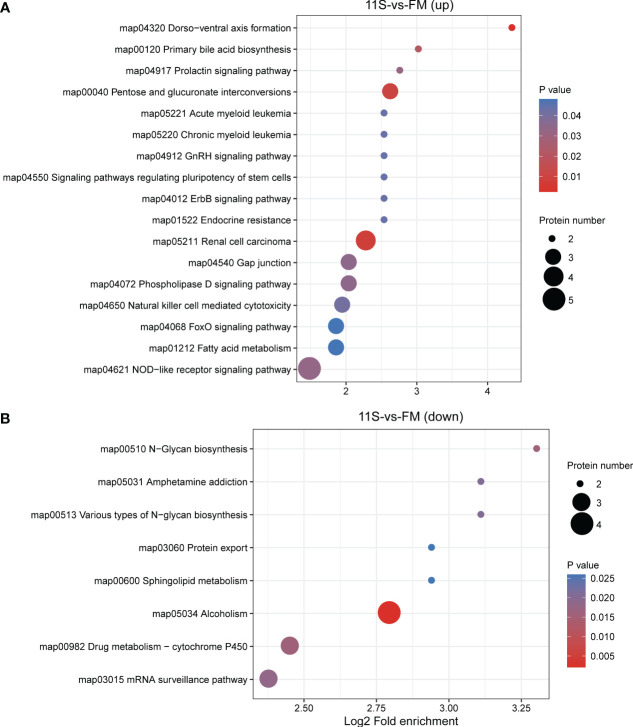
Enrichment analysis of KEGG pathway for differentially expressed protein (DEPs) in 11S-vs-FM comparison group. Log2 Fold enrichment is displayed on the horizontal axis, while the vertical axis denotes KEGG pathway names. Bubble size signifies protein counts within each pathway. The enriched *P*-value is represented by a color. **(A)** Enrichment by upregulated DEPs. **(B)** Enrichment by downregulated DEPs.

In the 11S and FM comparison group, 169 DEPs were functionally annotated using KEGG analysis. Among these, upregulated proteins showed significant enrichment pathways related to immune system- and human disease, such as NOD-like receptor signaling pathway, natural killer cell-mediated cytotoxicity, chronic myeloid leukemia (ko05220), renal cell carcinoma (ko05211), and acute myeloid leukemia (ko05221, P *< 0.05*, **Figure 5A**). Down-regulated proteins showed significant enrichment in pathways, such as alcoholism (ko05034), N-glycan biosynthesis (ko00510), drug metabolism-cytochrome P450 (ko00982), mRNA surveillance pathway (ko03015), various types of N-glycan biosynthesis (ko00513), amphetamine addiction (ko05031), sphingolipid metabolism (ko00600), and protein export (ko03060*;* P *< 0.05*, **Figure 5B**).

**Figure 6:**
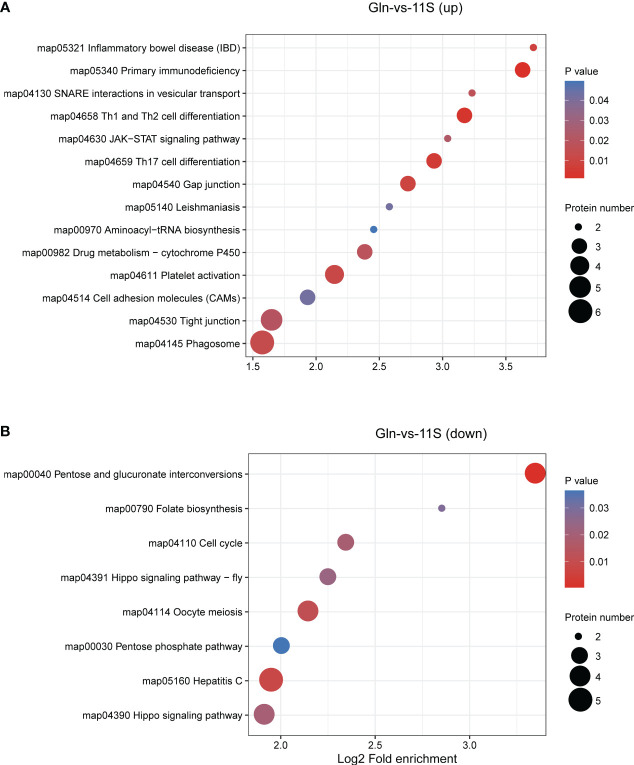
Enrichment analysis of KEGG pathway for differentially expressed protein (DEPs) in Gln-vs-11S comparison group. Log2 Fold enrichment is displayed on the horizontal axis, while the vertical axis denotes KEGG pathway names. Bubble size signifies protein count within each pathway. The enriched *P*-value is represented by color. **(A)** Enrichment by upregulated DEPs. **(B)** Enrichment by downregulated DEPs.

In the Gln and 11S comparison group, up-regulated proteins showed significant enrichment in pathways associated with immune system- and human disease, such as Th1 and Th2 cell differentiation (ko04658), platelet activation (04611), JAK-STAT signaling pathway (ko04630), Th17 cell differentiation (ko04659), primary immunodeficiency (ko05340), inflammatory bowel disease (IBD, ko05321), and leishmaniasis (ko05140; *P* < 0.05, **Figure 6A**). They also demonstrated significant enrichment in pathways associated with the intestinal epithelial barrier function, such as tight junction and cell adhesion molecules (*P*<0.05). Down-regulated proteins showed significant enrichment in pathways, such as oocyte meiosis (ko04114), pentose and glucuronate interconversions (ko00040), hippo signaling pathway (ko04391), hepatitis C (ko05160), cell cycle (ko04110), folate biosynthesis (ko00790), and pentose phosphate pathway (ko00030; P *< 0.05*, **Figure 6B**).

### Integration analysis of the DEGs and DEPs

**Figure 7:**
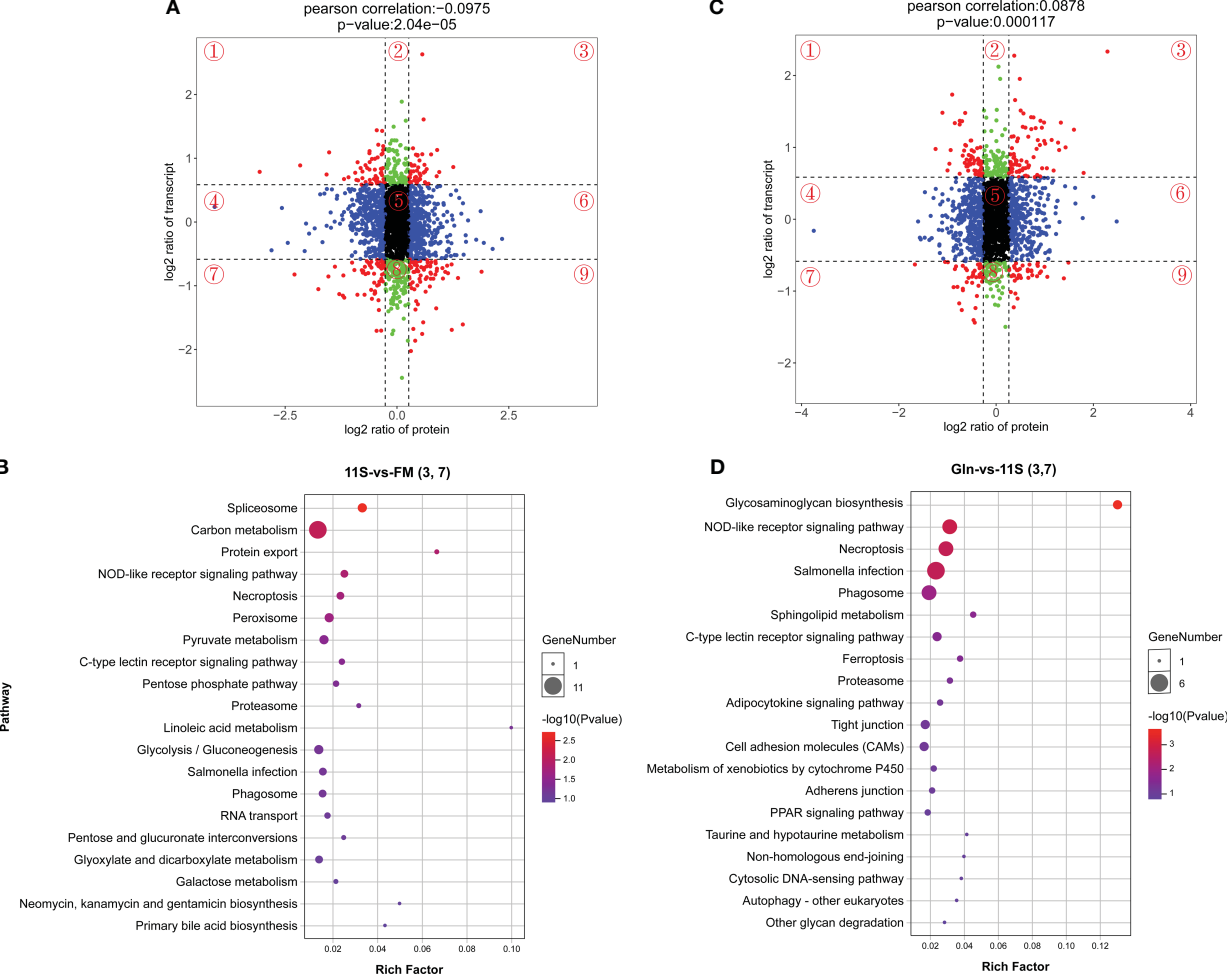
Top 20 KEGG enrichment analysis with differential mRNAs consistent with the corresponding differential protein expression (quadrants 3 and 7) in both the 11S-vs-FM and Gln-vs-11S group. The horizontal axis **(A, C)** represents the log2 ratio of protein, and the vertical axis **(A, C)** denotes the log2 ratio of transcript. The horizontal axis **(B, D)** indicates the rich factor, and the vertical axis **(B, D)** denotes the name of the KEGG pathway. Bubble size **(B, D)** indicates protein counts within each pathway. Enriched-log 10 *P*-value is represented by a color (**B, D)**.

We performed a nine-quadrant plot classification of DEGs and DEPs (**Figures 7A, C**), quadrants 1 and 9 indicate that the mRNA is inconsistent with the corresponding protein differential expression pattern; quadrants 2 and 8 indicate that the mRNA is differentially expressed and the corresponding protein is unchanged; quadrants 3 and 7 suggest concordance between mRNA and corresponding protein differential expression; quadrant 4 and 6 indicate differential expression of protein and no change in corresponding mRNA; quadrant 5 indicates that both co-expressed mRNA and protein are non-differentially expressed. Then, KEGG enrichment pathway analysis was performed on differentially expressed mRNAs and proteins consistent with quadrants 3 and 7 in the 11S and FM comparison group (**Figure 7B**). The results showed that spliceosome (ko03040), NOD-like receptor signaling pathway, carbon metabolism (ko01200), protein export, necroptosis, pyruvate metabolism (ko00620), C-type lectin receptor signaling pathway and pentose phosphate pathway were significantly enriched in the TOP 20 pathways (P < 0.05). Similarly, KEGG enrichment pathway analysis of differential mRNAs and proteins consistently expressed in quadrants 3 and 7 in the Gln and 11S comparison group showed that pathways such as glycosaminoglycan biosynthesis (ko00532), NOD-like receptor signaling pathway, necroptosis, phagosome, sphingolipid mem, C-type lectin receptor signaling pathway, and ferroptosis and proteasome showed significant enrichment (P *< 0.05*, **Figure 7D**). Furthermore, the leading 20 KEGG pathways showed enrichment in immune system-related pathways, including NOD-like receptor signaling pathway and phagosome, along with intestinal barrier-related pathways including tight junction, adherens junction, and cell adhesion molecules.

### mRNAs and proteins associated with the intestinal epithelial barrier

Myosin-1, Tubulin alpha-2, Actin (alpha skeletal muscle B), Major histocompatibility complex class II (MHC-II), Mucin-3B, Mucosal pentraxin, Leiomodin-1, Cytoplasmic dynein 1 heavy chain 1, Lysozyme, Eukaryotic translation initiation factor 5B, and Pyruvate kinase were significantly downregulated at both mRNA and protein levels in Group 11S than in Group FM (P *< 0.05*, **Table 2**). In the Gln and 11S comparison group, Ras-related C3 botulinum toxin substrate 2, Myosin-1, Cortactin, Wiskott-Aldrich syndrome protein, tenascin, Cluster of differentiation 4 (CD4), MHC-I, MHC-I -II, lysozyme, and NF-kappa-B inhibitor showed significant upregulation at both mRNA and protein levels (P *< 0.05*).

**Table 2:** Differential genes and proteins associated with the intestinal epithelial barrier in quadrants 3 or 7.

Groups	Gene/Protein name	log2FC.x	log2FC.y	Group	Pathway
11S vs FM	*myh11*/Myosin-11	-0.76	-0.79	7	Tight junction
	*α-tub*/Tubulin alpha-2	-0.67	-0.62	7	Tight junction
	*acta1b*/Actin, alpha skeletal muscle B	-0.33	-0.71	7	Tight junction
	*mhc-II*/Major histocompatibility complex class II	-0.26	-0.73	7	Cell adhesion molecules (CAMs)
	*muc3b*/Mucin-3B	-1.75	-1.05	7	ECM-receptor interaction
	*mptx*/Mucosal pentraxin	-1.20	-0.84	7	—–
	*lmod1*/Leiomodin-1	-0.37	-0.69	7	—–
	*dync1h1*/Cytoplasmic dynein 1 heavy chain 1	-0.33	-0.67	7	Phagosome
	*lys*/Lysozyme	-1.21	-1.16	7	NOD-like receptor signaling pathway
	*eif5b*/Eukaryotic translation initiation factor 5B	-0.45	-0.66	7	TOR signaling pathway
	*pk*/Pyruvate kinase	-0.35	-0.92	7	TCA cycle
Gln vs 11S	*myh11*/Myosin-11	0.40	0.82	3	tight junction
	*cttn1*/Cortactin	0.60	0.71	3	tight junction
	*tnc*/Tenascin	0.37	2.28	3	ECM-receptor interaction/Focal adhesion
	*cd36*/Cluster of differentiation 4	0.91	0.60	3	ECM-receptor interaction
	*mhc-I*/Major histocompatibility complex class I	0.39	1.66	3	Cell adhesion molecules (CAMs)
	*mhc-II*/Major histocompatibility complex class II	0.51	1.51	3	Cell adhesion molecules (CAMs)
	*ptprc*, *cd45*/Receptor-type tyrosine-protein phosphatase C	0.84	0.62	3	Cell adhesion molecules (CAMs)
	*lys*/Lysozyme	2.29	2.33	3	NOD-like receptor signaling pathway
	*iκB*/NF-kappa-B inhibitor	0.30	0.90	3	NF-κB signaling pathway

The fold change (FC) thresholds for the transcriptome and proteome were ≥1.5 and ≥1.2, respectively. Log2FC.x represents proteins and log2FC.y represents genes.

### miRNAs and their target genes involved in the intestinal epithelial barrier

**Figure 8:**
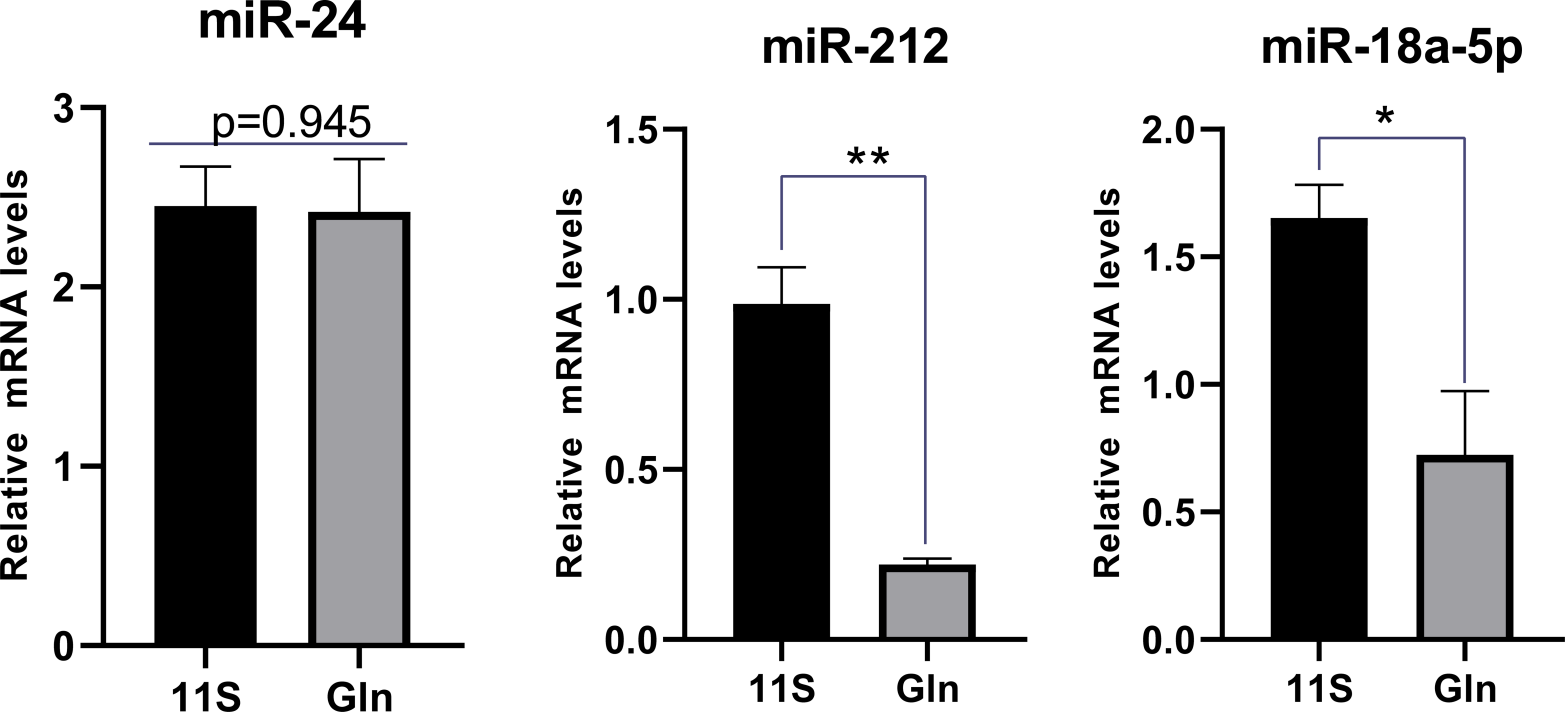
Targeted miRNA levels were analyzed by quantitative PCR (qPCR) in 11S and Gln groups. Screening of miRNAs regulating key genes associated with the intestinal barrier pathways based on a small RNA sequencing database in hybrid groupers. *0.01<P<0.05 and ** 0.001<P<0.01.

We also focused on genes with inconsistent mRNA and corresponding protein differential expression patterns and further screened the genes and proteins associated with the intestinal epithelial barrier function in the first quadrant (**Table 3**). MHC-I showed an upregulation at the mRNA level and a downregulation at the protein level in the 11S and FM comparison group. As shown by the miRNA target gene profile, miR-143_2, miR-222, miR-192-3p_2, miR-34a-5p_2, and miR-21b_3p were able to target the *mhc-I* gene. Similarly, the Gln and 11S comparison group found that Colla1 and Col1a2 exhibited an upregulation at the mRNA level and a downregulation at the protein level. Moreover, miR-24, miR-212, and miR-18a-5p were able to target the *colla1* gene, and miR-205a, miR-29a-3p, and miR-212 were able to target the *colla2* gene. In addition, the expression levels of miR-18a-5p and miR-212 in the intestine of the Gln group were notably lower than those in Group 11S (P *< 0.05*, **Figure 8**), while miR-24 expression between Groups 11S and Gln showed no significant difference (P *> 0.05).*

**Table 3:** miRNAs targeting genes and proteins related to the intestinal barrier function.

Groups	miRNA	Target Gene/Protein name	log2FC.x	log2FC.y	Group
11S vs FM	miR-143_2	*mhc-I*/ Major histocompatibility complex class I	-0.36	0.77	1
	miR-222				
	miR-192-3p_2				
	miR-34a-5p_2				
	miR-21b-3p				
Gln vs 11S	miR-24	*colla1*/Collagen alpha-1(I)	-0.75	1.37	1
	miR-212				
	miR-18a-5p				
	miR-205a	*col1a2*/Collagen alpha-2(I)	-0.85	1.34	1
	miR-29a-3p				
	miR-212				

Log2FC.x represents proteins and log2FC.y represents genes.

### Transcriptome and proteome validation

To validate the precision of the transcriptome findings of FM, 11S, and Gln groups. Ten genes (5 upregulation and 5 downregulation) were selected for qPCR validation in this experiment (**Supplementary Figure 3**). The agreement between RT-qPCR and transcriptome sequencing results underscores the enhanced accuracy of transcriptome sequencing. To validate the precision of the results of the three proteome groups (FM, 11S, and Gln), the DEPs validation analysis was then performed by PRM quantitative proteomics (**Supplementary Figure 4**). The results showed that the ribosomal protein L32 (RPL32), ribosomal protein S7 (RPS7), macrophage migration inhibitory factor (MMIF), malate dehydrogenase (MDH), and beta-hydroxysteroid dehydrogenase (β-HSD) proteins in the 11S and FM comparison group showed consistent expression levels between PRM and 4D-LFQ analyses (**Supplementary Figure 4A**). Moreover, the expression levels of CD45, RPL19, histone, annexin, and annexin max3 proteins in the Gln and 11S comparison group were consistent with the results of the 4D-LFQ analysis (**Supplementary Figure 4B**).

## Discussion

We previously found that dietary Gln improved growth performance and alleviated intestinal inflammation induced by glycinin in hybrid grouper juveniles (27). However, the potential protective mechanism by which Gln alleviates enteritis in hybrid grouper remains unclear. On this basis, we further revealed its protective mechanism against soybean glycinin-induced hybrid grouper enteritis by integrating transcriptomic, proteomics, and miRNAs analyses. In the 11S and FM comparison group, the foremost 20 KEGG pathways involved in the immune system- and disease processes-related pathways, such as phagosomes and herpes simplex infection, as well as involved in the intestinal epithelial barrier-related pathways, such as tight junction, focal adhesion, apoptosis, and necroptosis, were significantly enriched. Analogous pathways have been identified in carnivorous fish that experience SBMIE, such as Atlantic salmon (*Salmo salar*) (10, 36) and turbot (37, 38). We also reported that these pathways above in hybrid groupers were enriched in the soybean meal substituted 50% of fishmeal (SBM50) and fishmeal comparison group (39). In addition, the downregulated genes were involved in intestinal epithelial barrier-related pathways such as tight junction, focal adhesion, ECM-receptor *interaction*, and cell adhesion molecules (CAMs), suggesting impaired intestinal development and increased intestinal permeability in fish fed 11S diet alone. When Gln was added to the 11S diet, the upregulated genes exhibited a pronounced enrichment in pathways associated with the immune system. These included the toll-like receptor signaling pathway, NOD-like receptor signaling pathway, C-type lectin receptor signaling pathway, RIG-I-like receptor signaling pathway, intestinal immune network for IgA production, and MAPK signaling pathway. Furthermore, there was significant enrichment in pathways related to the intestinal epithelial barrier, including focal adhesion, ECM-receptor interaction, tight junction, regulation of actin cytoskeleton, and cell adhesion molecules (CAMs). The above results suggested that Gln enhanced intestinal immune and intestinal epithelial barrier functions and reduced the occurrence of hybrid grouper enteritis induced by soybean 11S. Similar results have been observed in various fish species, showing that the addition of Gln in the feed was effective in alleviating the clinical symptoms of trinitrobenzene sulfonic acid-induced enteritis in grass carp (*Ctenopharyngodon idella*) (40) and soybean antigenic protein-induced enteritis in Jian carp (*Cyprinus carpio var* Jian) (9, 41), and in promoting intestinal barrier function and hindgut morphology of soybean meal-induced enteritis in turbot (30, 38).

Proteins are the direct function executors of myriad life activities. Proteomics enables population assessment of protein expression levels, composition, and modification status in samples through high-throughput analysis, which in turn reveals protein functions, potential relationships between proteins, and the mining of new proteins. The proteomics data of this study showed that a total of 169 DEPs were found in the comparison of the 11S and FM group, and 165 DEPs were found in the Gln and 11S comparison group. These DEPs of the two comparison groups were mainly distributed in the cytoplasm, with a percentage of 46.15% and 46.67%, respectively, suggesting that the DEPs may mainly play important functions in the cytoplasm. In addition, KEGG functional annotation was performed on these DEPs. In the Gln and 11S comparison group, the expression of upregulated proteins displayed significant enrichment in pathways associated with the immune system and human disease pathways, including NOD-like receptor signaling pathway, natural killer cell-mediated cytotoxicity, renal cell carcinoma, chronic myeloid leukemia, and acute myeloid leukemia, implying that these signaling pathways above may play an important role in glycinin-induced enteritis in hybrid groupers. When soybean 11S feeds were supplemented with Gln, the up-regulated DEPs showed significant enrichment in intestinal epithelial barrier-related pathways including tight junction and cell adhesion molecules (CAMs). Similar results have been observed in the jejunum of maternal and piglets, demonstrating that dietary Gln increased the translation levels of intestinal tight junction and cell adhesion molecule proteins (42). Notably, immune system- and human disease-related pathways, including Th1 and Th2 cell proliferation, Th17 cell proliferation, platelet activation, JAK-STAT signaling pathway, primary immunodeficiency, inflammatory bowel disease, and leishmaniasis were also significantly enriched in Gln and 11S comparison group, suggesting a close link between intestinal epithelial barrier function and immune system pathways in hybrid grouper with Gln supplementation in soya 11S feed.

Correlation analysis of transcriptomic and proteomics data offers a more complete insight compared to single omics, and the two can mutually validate the reliability of the data. In this study, 2,057 genes were associated with the mRNA and protein levels in the FM, 11S, and Gln groups. The correlation coefficients of gene expression associated with the 11S-vs-FM and Gln-vs-11S comparison groups at the mRNA and protein levels were -0.10 and 0.09, respectively, indicating that the mRNA-protein correlation in this study was low. Huang et al. (43) correlated the transcriptome and proteome of *Cyanobacteria* at two points of time (24 h and 48 h) under nitrogen starvation and found correlation coefficients of 0.04 and -0.001, respectively. The process of translation from mRNA to protein is subject to complicated regulation, such as post-transcriptional regulation and protein translation modification, resulting in a weak correlation between transcriptome and proteome (44). In order to clarify the mechanism of protective effect of Gln in alleviating soybean 11S-induced grouper enteritis, differential expressed mRNAs and consistently expressed proteins were further analyzed for KEGG enrichment pathway. Genes such as *myosin-1*, *tubulin alpha-2*, *alpha-actin*, *major histocompatibility complex class II* (*mhc-II*), *mucin-3B*, *mucosal pentraxin*, *leiomodin-1*, *cytoplasmic dynein 1 heavy chain 1*, *lysozyme*, and *eukaryotic translation initiation factor 5B* were down-regulated at both mRNA and protein levels in the 11S and FM comparison group. In addition, *myosin-1*, *cortactin*, *Wiskott-Aldrich syndrome protein*, *ras-related C3 botulinum toxin substrate 2*, *tenascin*, *cd4*, *mhc-I*, *mhc-II*, *lysozyme*, and *iκBα* were upregulated at both the transcriptional and translational levels in the Gln and 11S comparison group. These genes participate in intestinal epithelial barrier pathways, including tight junction, adhesion junction, cell adhesion molecules (CAMs) and ECM-receptor interaction, as well as NOD-like receptor signaling pathway and NF-κB signaling pathway. Tight junctions are essential for animal organisms to establish a selective permeability barrier between neighboring cells. Myosin in tight junctions is the most important component of fish muscle proteins responsible for the contractile function of myogenic fibers and has ATPase activity, which binds actin and forms fibers under physiological conditions of low ionic strength (45). In addition, cortactin is associated with a variety of complex cellular processes, including cell motility, invasiveness, synaptogenesis, phagocytosis, tumorigenesis, and metastasis formation (46). Overexpression of cortactin could contribute to the emergence of invasive tumor phenotypes in a variety of ways, including enhanced actin polymerization, down-regulated epidermal growth factor receptor, and molecule interactions between cyclin D1 and CD44 proteins (46). The extracellular matrix (ECM) is a complex blend of structural and functional macromolecules, which hold a vital role in the development of tissues and organs, and the preservation of cells and tissue (47). The tenascin involved in the ECM was also reported in Gln on mouse mesangial cells (48), showing that the mRNA expression level of tenascin was not affected after treating the cells with 2 mM Gln compared to the control group (no Gln was added), which was inconsistent with the results of the present experiments, probably due to the differences in the different species, *in vitro* and *in vivo* experiments. After infection of bone marrow-derived macrophages (BMDMs) using the bacterium *Leishmania donovani*, supplementation with Gln can significantly increase the gene expression of *mhc-II* (49), which is similar to the results of the present experiment. The MHC also comprises the most polymorphic genes in the vertebrate genomes that are closely related to immune response (50). IκB is an inhibitor of NF-κB, and NF-κB activity is inhibited when it is present. The IKK complexes, encompassing IKKα, IKKβ, and IKKγ, are capable of initiating the phosphorylation of IκB. The phosphorylation event prompts the degradation of IκB, subsequently culminating in the activation of NF-κB. This activation involves various subunits of NF-κB, including NF-κB p52, NF-κB p65, and c-Rel. As a result, there is an up-regulation in the expression of pro-inflammatory cytokines like tnf-α (51). The present study and our previous results (27) also found that dietary Gln down-regulated *ikkβ*, *nf-κb*, *tnf-α*, *il-1β*, *ifn-α*, and *hsp70* mRNA expression levels as well as up-regulated IκB expression at both mRNA and protein levels and ultimately reduced the occurrence of inflammation in hybrid groupers. Similar results were found for another amino acid, Met-Met, showing that suitable dietary Met-Met down-regulated the gene levels of *nf-κb p65*, *c-rel*, i*kkβ*, and *ikkγ* and up-regulated the gene levels of *iκBα* in the intestinal tract of juvenile grass carp (52). In addition, lysozyme can remove the residual cell wall after the action of antibacterial factors, enhance the antibacterial sensitivity of other immune factors, synergize with other immune factors to resist the invasion of foreign pathogens, which increases the activity of serum lysozyme and improve its immunity accordingly (53). Our previous results also showed that soybean 11S reduced the intestinal lysozyme activity of hybrid grouper at both the transcriptional and protein levels, whereas the supplementation of Gln in the 11S feed increased the lysozyme activity at both mRNA and protein levels. This suggests a potential enhancement in the intestinal immune function of the hybrid grouper.

The primary factor contributing to the limited correlation between transcriptome and proteome data arises from the intricate regulation occurring at multiple stages of gene expression. This includes transcription of DNA into mRNA and subsequent translation of mRNA into protein. Diverse factors exert control over these processes, encompassing both transcriptional and translational levels, as well as post-translational modifications. These multifaceted regulatory mechanisms lead to variations in mRNA transcript numbers, protein localization, abundance, and functionality. Consequently, these dynamic changes disrupt the alignment between mRNA and its corresponding protein, resulting in the observed reduced correlation between the two. We next focused on the role of miRNAs in post-transcriptional and translational control of gene expression from the miRNA level (16) to further explain genes that are inconsistent at the mRNA and protein levels. The intestinal miRNA expression profile and their target genes were obtained from the previous experiment, showing that the *mhc-I* gene (up-regulated at transcriptional level and down-regulated at translational level) could be regulated by miR-143_2, miR-222, miR-192-3p_2, miR-34a-5p_2, and miR-21b-3p. The result implied that these miRNAs likely have a significant role in regulating the target mRNA/protein (MHC-I). In addition, the target genes of miR-24, miR-24-3p, miR-18a-5p, and miR-212 in the Gln and 11S comparison group were *type I collagen α1*; the target genes of miR-205a, miR-29a-3p, and miR-212 were *type I collagen α2*. Collagen has strong biological activity and function and plays a crucial role in mediating cell migration, differentiation, and proliferation (54). In this experiment, *collagen α1* and *collagen α2 genes* were up-regulated at transcriptional level and down-regulated at translational level, suggesting that these miRNAs above may inhibit the translational level of *collagen α1* and *collagen α2* genes. The qPCR results of miRNA further confirmed that miR-18a-5p and miR-212 expression levels were significantly affected in the Gln and 11S groups. Notably, the down-regulation of miR-212 expression targeted *collagen α1* and *collagen α* genes. Our early miRNA data showed that miR-212 had significantly higher expression levels (log_2_FC=2.182) in the SBM50 and FM comparison group (39). MiR-212 is a potent therapeutic target in mouse intestinal epithelial cells, where it affects a variety of T cells. Inhibition of miR-212/132 led to the induction of Treg1 and CD4+ cells and caused a decrease in Th17 cells (55). During chronic HIV/SIV infection, the disrupted expression of miR-212 in colonic epithelial cells can contribute to the disruption of the epithelial barrier by down-regulating the expression of occludin and PPARγ (56). The increased expression levels of Collagen α1 and Collagen α2 proteins, miR-18a-5p and miR-212 will be a key point. Notably, further validation of the targeting relationship of these miRNAs with target genes by dual luciferase reporter is needed.

In conclusion, enteritis induced by soybean glycinin was affected by mRNA and protein levels. By integrated transcriptome and proteome, 117 genes showed consistent expression patterns at both the transcriptional and translational levels in the Gln and 11S comparison group. Further found that the intestinal epithelial barrier pathways mediated the molecular mechanism in Gln alleviation of grouper enteritis induced by soybean glycinin. In addition, some miRNAs such as miR-212 and miR-18a-5 play key regulatory roles in Gln alleviation of hybrid grouper enteritis. Our findings provide valuable insights into the RNAs and protein profiles, contributing to a deeper understanding of the underlying mechanism for fish enteritis.

## Data availability statement

The datasets presented in this study can be found in online repositories. The names of the repository/repositories and accession number(s) can be found below: NCBI via accession ID PRJNA1008292 and ProteomeXchange Consortium via the iProX partner repository with the dataset identifier PXD044757.

## Ethics statement

The animal study underwent review and approval by the Animal Research and Ethics Committees of Guangdong Ocean University, China. The study was conducted in accordance with the local legislation and institutional requirements.

## Author contributions

YH: Methodology, Software, Writing – original draft, Writing – review & editing. XD: Writing – review & editing, Formal Analysis. QY: Formal Analysis, Writing – review & editing. SZ: Formal Analysis, Writing – review & editing. HL: Formal Analysis, Writing – review & editing. SX: Formal Analysis, Writing – review & editing. SC: Writing – review & editing, Conceptualization, Funding acquisition. BT: Conceptualization, Funding acquisition, Writing – review & editing.
