# Supplementary material for: An integrated study of glutamine alleviates enteritis induced by glycinin in hybrid groupers using transcriptomics, proteomics and microRNA analyses
Source: Front Immunol. 2023 Nov 22;14:1301033. doi: 10.3389/fimmu.2023.1301033 (PMC10702536; doi:10.3389/fimmu.2023.1301033)
Supplement: Supplementary file 2 [file DataSheet_2.docx]

**Supplementary documents**

**Tables**

**Supplementary Table 1.** Primer information of miRNAs and mRNAs for RT-qPCR

| Gene ID | Primer sequence (5’-3’) | Product size (bp) |
| --- | --- | --- |
| miR-210-5p | AGCCACTGACTAACGCACAT |  |
| miR-24 | TGGCTCAGTTCAGCAGGAACAGG |  |
| miR-34a-5p_2 | TGGCAGTGTCTTAGCTGGTTGTT |  |
| U6 | F: GGAACGATACAGAGAAGATTAGC |  |
|  | R: TGGAACGCTTCACGAATTTGCG |  |
| CL1058.Contig1_All | F: TGGTCTCTCCGTCTGTTCCA | 115 |
|  | R: CAGAAAGCAAACAGCGGCAA |  |
| CL4098.Contig3_All | F: ACCTTCGGGAACGAAGTCAC | 208 |
|  | R: TATGAACCAGCTGCCAAGCA |  |
| CL1176.Contig3_All | F: CGATACCGTAGCCTCCACAC | 215 |
|  | R: GCTAGGGCCCATGAGGTTTT |  |
| CL3000.Contig4_All | F: TGTCCTAGTTTGCTGTCTCTGT | 102 |
|  | R: ACAGTGAGTTTTTGGCACATAGC |  |
| CL2358.Contig3_All | F: CTCAACTGCTTATGGCGTGC | 92 |
|  | R: CGGAGGAAACCAGAATCGCT |  |
| CL1675.Contig1_All | F: GCTGGCCATGTTGTGTCCTA | 120 |
|  | R: CAAGGGACCCAGAAGTGGAC |  |
| CL374.Contig8_All | F: ACCGGAGATGGTGTAGTGGA | 129 |
|  | R: TGGCACAGACAGACGGTAAC |  |
| Unigene115_All | F: CTCGAGCCCAAGTCATGGTT | 137 |
|  | R: TGAACACGGGACAGTGGAAG |  |
| CL1132.Contig5_All | F: TGTTAGCAGGGGCATTCGTT | 198 |
|  | R: TTGGACGGATGTTTGGGGAC |  |
| Unigene14461_All | F: TTTGCCGTCATCGGTCAGTT | 177 |
|  | R: TATGGCTGGAAGCCTGTGTG |  |
| β-Actin | F: GGCTACTCCTTCACCACCACA | 188 |
|  | R: TCTGGGCAACGGAACCTCT |  |

**Figures**


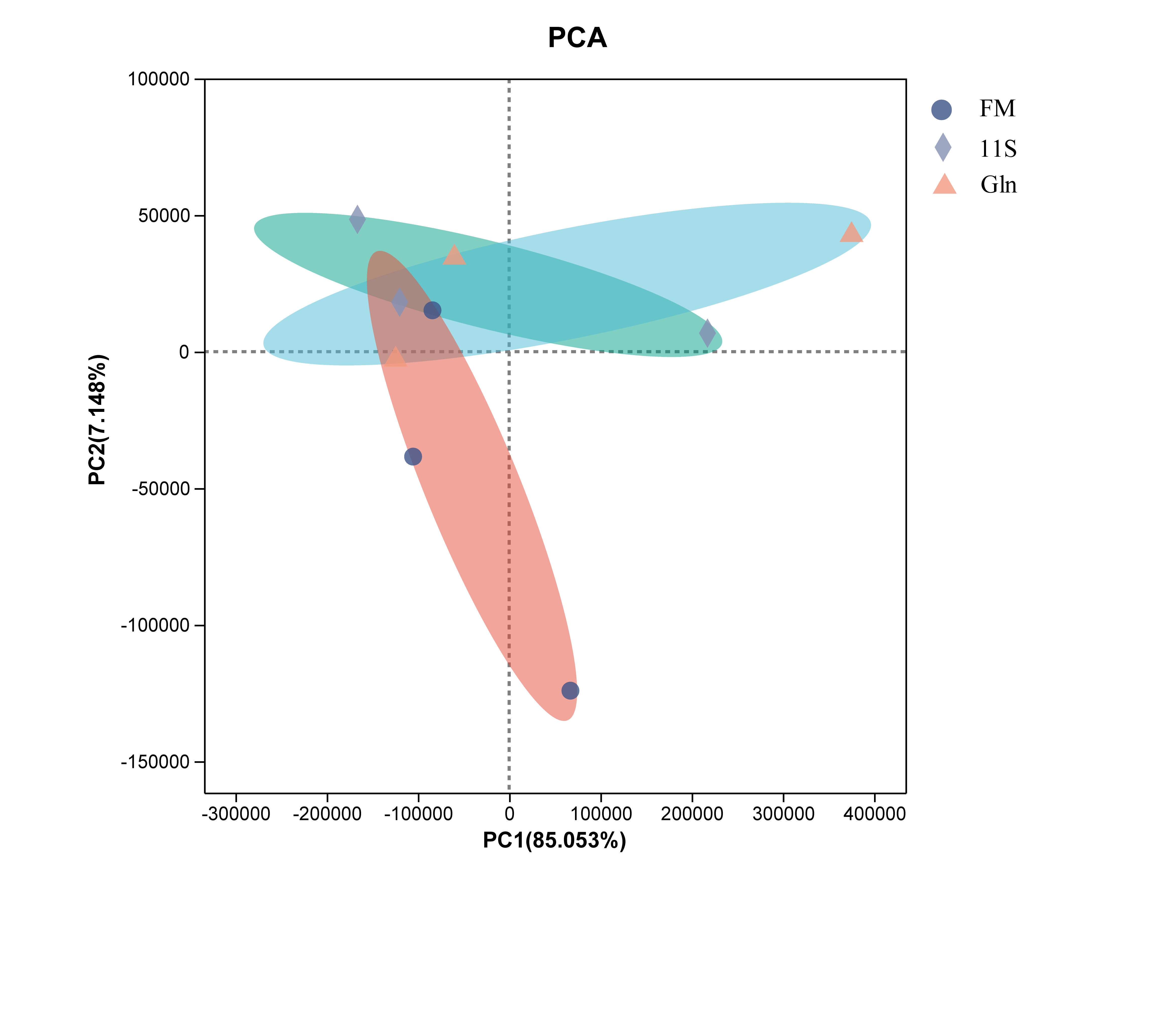


**Supplementary Figure 1.** A two-dimensional principal component analysis (PCA) plot was performed to assess the variability of samples within and between groups. The plot of the first two axes from a PCA based on counts of the gene across all samples FM (*n* = 3), 11S (*n* = 3), and Gln (*n* = 3)


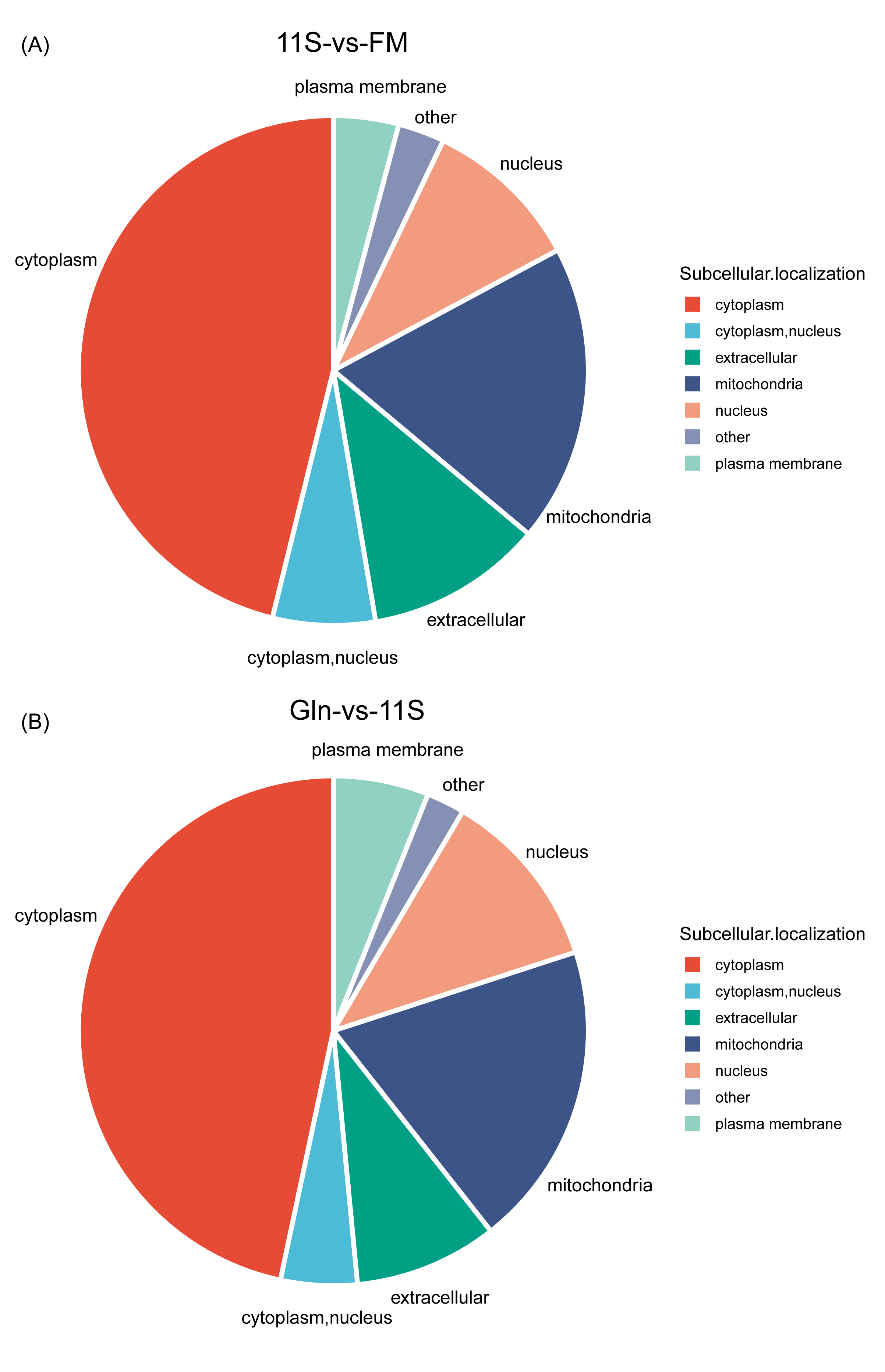


**Supplementary Figure 2.** Subcellular localization of the differentially expressed protein in both 11S-vs-FM and Gln-vs-11S comparison groups

**Supplementary Figure 3.** Comparison of RNA Seq and RT-q PCR results in both 11S-vs-FM (A) and Gln-vs-11S comparison groups (B)


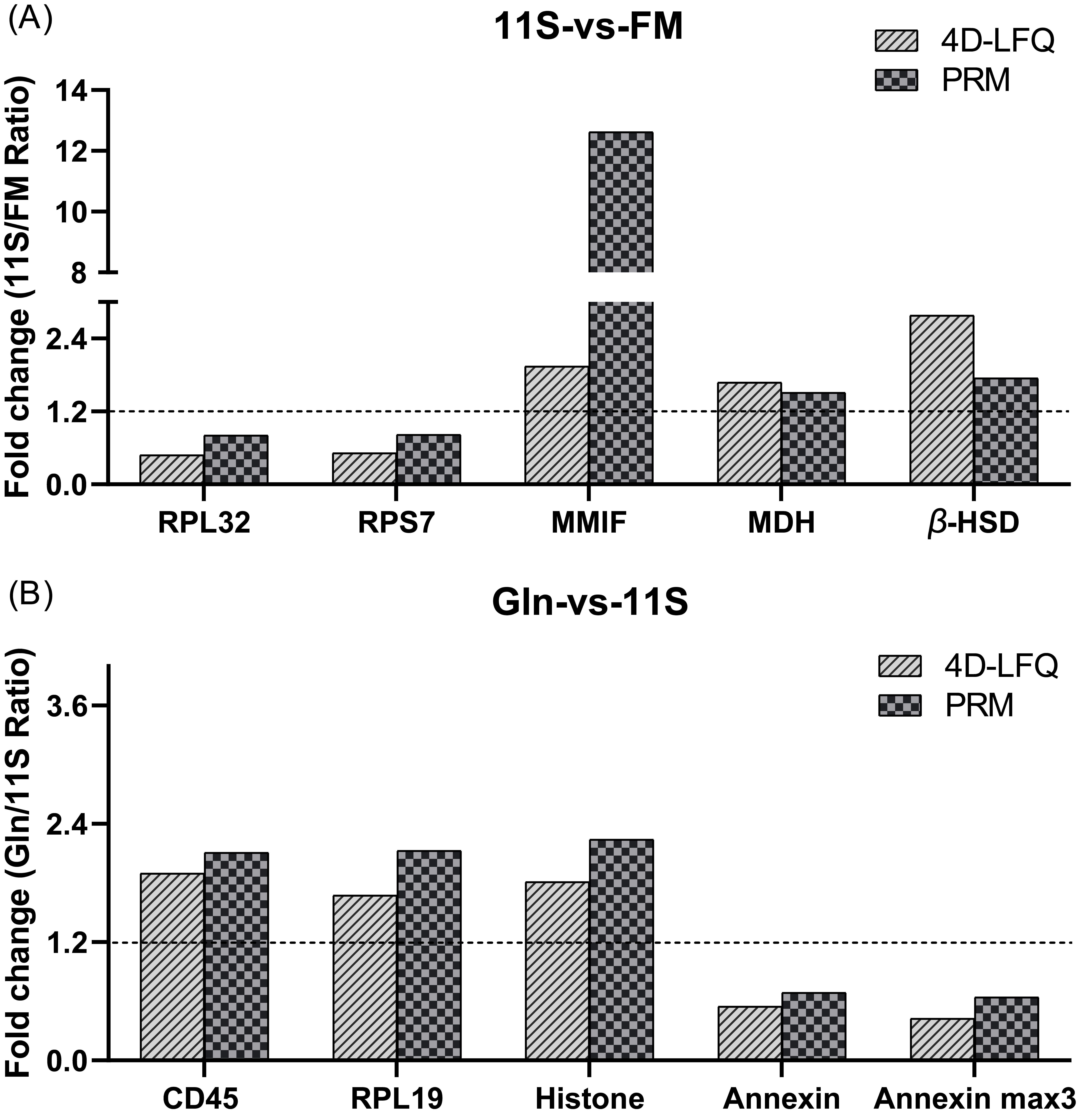


**Supplementary Figure 4.** The comparison of protein expression by 4D-LFQ and PRM. (A) protein expression in the 11S-vs-FM comparison group; (B) protein expression in the Gln-vs-11S comparison group. HSP10, heat shock protein 10; RPL32, ribosomal protein L32; MMFI, macrophage migration inhibitory factor; RPS7, ribosomal protein S7; MDH, malate dehydrogenase; β-HSD, beta-hydroxysteroid dehydrogenase; RPL19, ribosomal protein L19.
